# High survival and treatment success sustained after two and three years of first-line ART for children in Cambodia

**DOI:** 10.1186/1758-2652-13-11

**Published:** 2010-03-21

**Authors:** Petros Isaakidis, Marie-Eve Raguenaud, Vantha Te, Chhraing S Tray, Kazumi Akao, Varun Kumar, Sopheak Ngin, Eric Nerrienet, Rony Zachariah

**Affiliations:** 1 Médecins Sans Frontières, Phnom Penh, Cambodia; 2 Pediatrics Department, Donkeo Referral Hospital, Ministry of Health, Takeo, Cambodia; 3 Angkor Hospital for Children, Siem Reap, Cambodia; 4 HIV/Hepatitis Laboratory, Pasteur Institute of Cambodia; 5 Médecins Sans Frontières, Operational Research Unit, Brussels, Belgium

## Abstract

**Background:**

Long-term outcomes of antiretroviral therapy (ART) in children remain poorly documented in resource-limited settings. The objective of this study was to assess two-and three-year survival, CD4 evolution and virological response among children on ART in a programmatic setting in Cambodia.

**Methods:**

Children treated with first-line ART for at least 24 months were assessed with viral load testing and genotyping. We used Kaplan-Meier analysis for survival and Cox regression to identify risk factors associated with treatment failure.

**Results:**

Of 1168 registered HIV-positive children, 670 (57%) started ART between January 2003 and December 2007. Survival probability was 0.93 (95% CI: 0.91-0.95) and 0.91 (95% CI: 0.88-0.93) at 24 and 36 months after ART initiation, respectively. Median CD4 gain for children aged over five years was 704 cells/mm^3 ^at 24 months and 737 at 36 months. Median CD4 percentage gain for children under five years old was 15.2% at 24 months and 15% at 36 months. One hundred and thirty children completed at least 24 months of ART, and 138 completed 36 months: 128 out of 268 (48%) were female. Median age at ART initiation was six years.

Overall, 22 children had viral loads of >1000 copies/ml (success ratio = 86% on intention-to-treat-analysis) and 21 of 21 presented mutations conferring resistance mostly to lamivudine and non-nucleoside reverse transcriptase inhibitors. Risk factors for failure after 24 and 36 months were CD4 counts below the threshold for severe immunosupression at those months respectively. Only two out of 22 children with viral loads of >1000 copies/ml met the World Health Organization immunological criteria for failure (sensitivity = 0.1).

**Conclusions:**

Good survival, immunological restoration and viral suppression can be sustained after two to three years of ART among children in resource-constrained settings. Increased access to routine virological measurements is needed for timely diagnosis of treatment failure.

## Background

Pediatric HIV/AIDS care programmes in resource-limited settings have been reporting good antiretroviral treatment (ART) outcomes, comparable to those in high-income countries [1-15]. However, most of these reports describe early experiences and treatment outcomes. Reports of medium- and long-term clinical and immunological outcomes among children on ART in resource-poor settings are still scarce and, when available, are usually limited to relatively small cohorts. Similarly, there are few studies of medium- and long-term virological outcomes and antiretroviral drug resistance among children receiving ART in programmatic settings where viral load (VL) measurements and genotyping are seldom available [1,2].

With an estimated HIV prevalence of 0.9% in the adult population (15-49 years) by the end of 2008 (estimated at 2.2% in 1997) and an estimated 4400 children living with HIV, Cambodia is one of the worst-affected countries in south-east Asia [16,17]. Médecins Sans Frontières (MSF), the Cambodian National Center for Control of HIV/AIDS, Dermatology and STD (NCHADS), and Angkor Hospital for Children in Siem Reap have been treating HIV-positive children in Siem Reap and Takeo provinces since February 2003 and 2004, respectively.

More than 1100 HIV-positive children had been enrolled and more than 650 had been started on ART by the end of 2007. These two large-scale pediatric programmes represented 25% of the national pediatric HIV cohort and more than 25% of the total children on ART in the country. We previously described excellent outcomes, with more than 80% virological success in this pediatric cohort, after 12 months of treatment [4,5].

The aim of the current study was to assess whether the effectiveness of ART was sustained after 24 to 36 months of follow up. We report on survival, CD4 count evolution, virological response, and antiretroviral drug resistance patterns among HIV-positive children receiving first-line ART in a programmatic setting in Cambodia.

## Methods

### Setting

In collaboration with NCHADS, MSF supported pediatric HIV programmes in two hospitals in Cambodia: the Angkor Hospital for Children, a charity hospital located in Siem Reap province (population 700,000), and Donkeo Referral Hospital, a public health facility in Takeo Province (population 800,000). Since 2003 and 2004, respectively, ART was offered free of charge to HIV-positive children, confirmed with serology or reverse transcriptase polymerase chain reaction (RT-PCR) for children under 18 months, and CD4 count below 20% for children younger than five years of age, or below 200 cells/mm^3 ^for children older than five years.

### Treatment and follow up

The standard first-line regimen was stavudine, lamivudine and nevirapine, as recommended by the World Health Organization (WHO) [18]. Zidovudine and efavirenz were used as alternatives in case of intolerance or interaction with other drugs.

Preparation for ART initiation included a minimum of three counselling sessions for the caregiver and patient, depending on the age of the child. Follow-up visits were monthly throughout the study period.

CD4 count and/or CD4 percentage were monitored every six months. Viral load monitoring and genotyping were not routinely performed, but only used to confirm clinical and/or immunological failure by treating physicians. Adherence support was provided to caregivers and children at each visit by specially trained counsellors through individual and age-specific support group sessions. An active tracing system for patients who failed to attend a clinic appointment was set up, and included home visits, additional counselling sessions and psychosocial support.

### Study population

All HIV-positive children aged 15 years or younger who had registered in the two pediatric HIV/AIDS programmes and who had completed at least 24 months of first-line ART by December 2007 were eligible for this study. All children ever started on ART, regardless of time on treatment, were included in the overall survival analysis of the total cohort and the cohort CD4 evolution analysis.

### Cross-sectional viral load measurements and genotyping

Between December 2007 and October 2008, eligible patients had a VL measurement done together with their routine CD4 test. CD4 measurements were performed at a Cambodian Ministry of Health Reference Laboratory (Kampong Cham Referral Hospital) and at the National Institute of Public Health Laboratory in Phnom Penh. HIV-1 RNA VLs were measured at the Pasteur Institute of Cambodia by real-time RT-PCR, using the ANRS G2 LTR-based real-time RT-PCR assay (Generic HIV-1 viral load, Biocentric, Bandol, France) [19]. For patients with VL >1000 HIV-1 RNA copies/ml of plasma, a genotypic resistance test was done. HIV-RNA was extracted (QIAampViral RNA mini kit, Qiagen, Germany). RT-PCR-amplified fragments in the reverse transcriptase gene of HIV-1 were sent to Macrogen Company (Macrogen Inc. Seoul, Republic of Korea) for sequencing.

Chromatograms, sent back electronically to Pasteur Institute of Cambodia, were verified, analyzed and interpreted. Clustal X 1.81 software was used for alignment with subtype reference sequences set from the Los Alamos HIV Sequence Database (http://www.hiv.lanl.gov/content/sequence/HIV/refer.html as well as for phylogenetic analysis using a nucleotide-distance matrix and the bootstrap neighbor-joining method. The resistant mutations were defined according to the WHO HIV Drug Resistance Database http://hivdb.stanford.edu/pages/WHOResistanceList.html and the drug susceptibility was predicted according to the French National Agency for AIDS Research (ANRS) algorithm http://www.hivfrenchresistance.org/.

In addition, for each study participant, a short questionnaire was completed by a counsellor or doctor on the same day that an informed consent was obtained. Questions were aimed at identifying factors potentially associated with poor adherence, such as: age of caregiver; education and literacy level of caregiver; distance of house to the clinic; size of family; and status of parents (alive/dead).

### Statistical analysis

Medical follow-up data were routinely collected at each consultation and entered prospectively into FUCHIA^© ^monitoring software (Follow up and Care of HIV Infection and AIDS, EPICENTRE, Paris).

The Kaplan-Meier method was used to estimate survival for all children on an intention-to-treat basis. Patients were censored on the date of their last visit, or date of transfer, or date of death, before 31 December 2007. CD4 gains were calculated on a six-monthly basis after ART initiation and weight-for-age z-score increases were recorded annually. Success ratio of the entire ART cohort was calculated on an intention-to-treat basis.

Treatment failures were defined as patients with VLs of >1000 copies/ml (before and after 24 months of ART), and patients who died, were lost to follow up, or were not assessed. Patients who did not return within three months of their scheduled follow-up visit were considered lost to follow up. Rate ratios were calculated to identify factors associated with virological failure after 24 months of ART. The Cox proportional hazards model was fitted to identify determinants of virological failure. Statistical analyses were performed using Stata 8.2 software (Stata Corporation, College Station, Texas, USA).

### Ethics

The study protocol was approved by the National Ethics Committee for Health Research of Cambodia, the Ethics Review Board of Médecins Sans Frontières, and the Institutional Review Board of Angkor Hospital for Children, Cambodia. All participants' caregivers gave their written informed consent.

## Results

### Outcomes and survival of all children on ART

**Figure 1:**
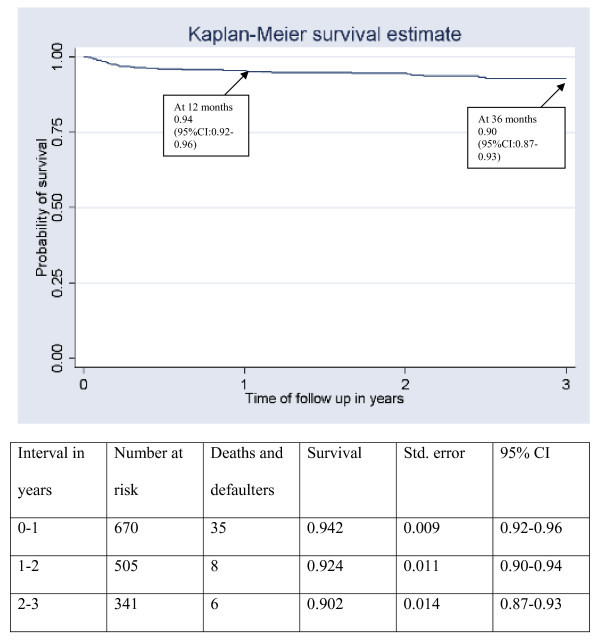
Survival of children who started ART, 2002-2008, Takeo and Siem Reap, Cambodia

A total of 1168 HIV-positive children were registered in the two HIV clinics between March 2002 and December 2007, of whom 670 (57%) started ART between January 2003 and December 2007. Among the 670 children who started ART, 37 (5.5%) had died, 13 (2.0%) were lost to follow up and 47 (7.0%) were transferred out by the end of December 2007. Median time of follow up since ART initiation for all children ever started ART was 24 months (IQR: 12-34). Estimates of survival after admission were 0.93 at 24 months (95% CI: 0.91-0.95) and 0.91 at 36 months (95% CI: 0.88-0.93) when death and loss-to-follow-up events were combined (Figure 1).

### Characteristics of children on ART ≥ 24 months at time of ART initiation

**Figure 2:**
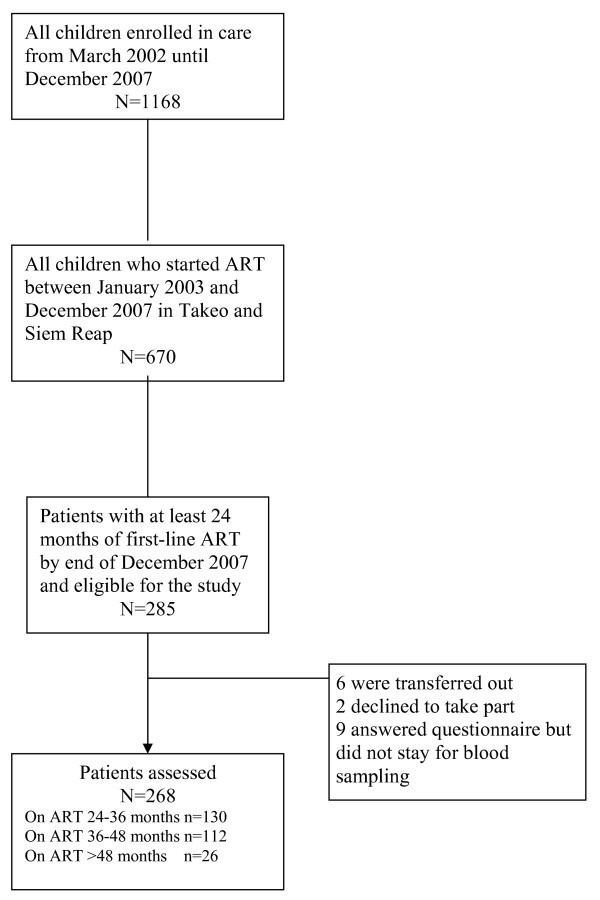
Flow chart of HIV-positive children on ART

Among the 670 children who had started ART, 285 were on first-line ART for at least 24 months by end of December 2007 and were thus eligible for the study (Figure 2). Among these, 268 were assessed with viral load measurement and completed the study questionnaire after 1 December 2007. Six patients were transferred out before study inclusion could take place, two declined to take part, and nine answered the questionnaire but did not stay for blood sampling. One hundred and thirty-eight of the 268 (51.5%) children were on ART for at least 36 months at the time of the assessment. Median follow-up time from ART initiation until end of observation time or date of failure was 36.2 months (IQR: 30.7-40.7).

One hundred and twenty-eight out of 268 (48%) were female and the median age was six years (IQR: 4-8). Only one child was aged less than 18 months at ART initiation. A high proportion of children were already at an advanced stage of HIV disease when starting treatment: 103 (38%) and 53 (20%) were at CDC stages B and C, respectively. Median CD4 count was 190 cells/mm^3 ^for children older than five years (IQR: 54-342), and median CD4 cell percentage for children under five years was 11.0% (IQR: 6.0-15.8) (Table 1). Regarding nutritional status at treatment initiation, the median weight-for-age z-score was -2.7 (IQR: -3.2 to -2.1).

**Table 1:** Characteristics at time of ART start of children on first line ART = 24 months

Characteristic	Value
All patients	268
Female, n (%), N = 268	128 (47.8%)
Age in years, median (IQR), N = 268	6 (4-8)
CDC stage at ART initiation, n (%), N = 268	
Stage N	22 (8.2%)
Stage A	90 (33.6%)
Stage B	103 (38.4%)
Stage C	53 (19.8%)
CD4 cell count/mm^3^, median (IQR) in children ≥60 months, N = 163	190 (54-342)
CD4 cell %, median (IQR) in children ≤59 months, N = 105	11.0 (6.0-15.8)
Weight-for-age z-score, median (IQR), N = 220	-2.65 (-3.2 to -2.1)
Initial antiretroviral treatment, n (%), N = 268	
3TC/D4T/NVP	208 (77.6%)
3TC/D4T/EFV	38 (14.2%)
3TC/AZT/NVP	22 (8.2%)
Time on ART in months, median (IQR), N = 268	36.2 (30.7-40.7)
Previous ART experience^a^, n	1
Number of patients who came late^b ^for a scheduled appointment at least once, n (%), N = 268	156 (58.2%)
Number of late consultations:	
0-1	192 (71.6%)
2-3	58 (21.6%)
≥4	18 (6.7%)
Geographic origin	
From the same province as the clinic, n (%)	122 (45.5%)
From another province, n (%)	146 (54.5%)
Status of parents	
At least 1 parent	171 (63.8%)
Orphan	95 (35.4%)
Unknown	2 (0.7%)
Education level of main caregiver	
No school	31 (11.6%)
Primary level	127 (47.4%)
Secondary level	108 (40.3%)
Unknown	2 (0.7%)
Literacy level of caregiver	
Literate	131 (48.9%)
Some literacy	66 (24.6%)
Illiterate	69 (25.7%)
Unknown	2 (0.7%)
Age of caregiver	
<30 years	57 (21.3%)
30-50 years	137 (51.1%)
>50 years	71 (26.5%)
Unknown	3 (1.1%)
Number of caregivers	
1	143 (53.3%)
>1	123 (45.9%)
Unknown	2 (0.7%)
Size of household	
2-5 people	136 (50.7%)
6-15 people	113 (42.2%)
>15 people	16 (6.0%)
Unknown	3 (1.1%)

Traveling distance to clinic	
≤ 1 hour	92 (34.3%)
>1 hour and 2 hours	51 (19.0%)
2 hours	123 (45.9%)
Unknown	2 (0.7%)

Receives food support	
Yes	187 (69.8%)
No	75 (28.0%)
Unknown	6 (2.2%)

^a ^Reported by patient or caregiver

^b ^Definition of "late": did not come on day of scheduled appointment

3TC: lamivudine, d4T; stavudine, AZT; zidovudine, NVP: nevirapine, EFV: efavirenz.

### Clinical and immunological outcomes of patients on ART ≥ 24 months

**Figure 3:**
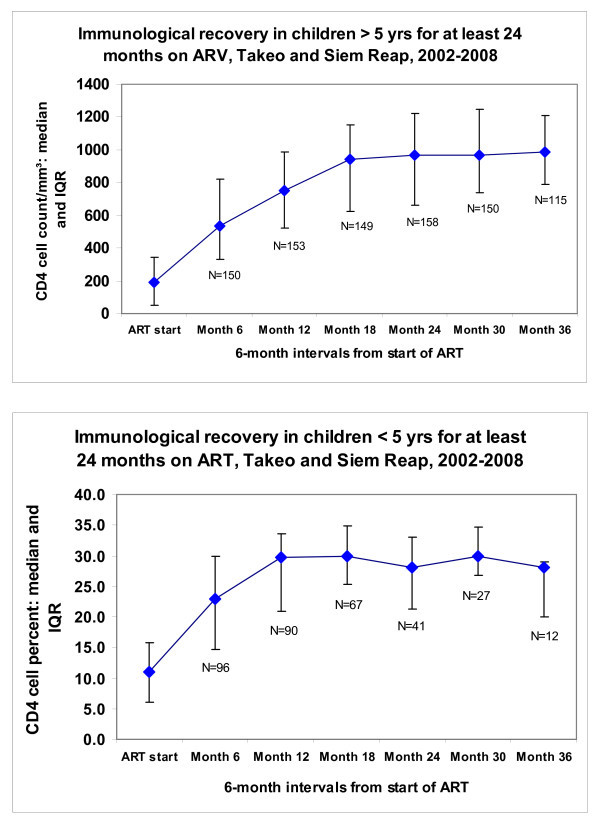
**Evolution of CD4 count/mm^3 ^and CD4% over time in pediatric cohort on first-line ART for at least 24 months**.

Median CD4 gain from baseline value for children over five years was found to be 704 cells/mm^3 ^(IQR: 469-1034) at 24 months (n = 158) and 737 (IQR: 546-924) at 36 months (n = 115) (Figure 2). Median CD4 percentage gain from baseline value for children under five years was found to be 15.2% (IQR: 12.6-21.6) at 24 months (n = 96) and 15.0% (IQR: 13.1-16.0) at 36 months (n = 12) (Figure 3). In the early years of the programmes, limited access to CD4 measurements resulted in some missing CD4 and CD4 percentage data in this cohort of children. The weight-for-age z-score median gain on ART was 1.88 (IQR: 1.41-2.43) during the second year, and 2.96 (IQR: 2.26-3.97) during the third year.

### Cross-sectional virological survey of patients more than 24 months on ART

Two hundred and forty out of 268 children assessed with VL had an undetectable VL (≤ 250 copies/ml), six had a VL between 250 and 1000 copies/ml and 22 had a VL above 1000 copies/ml, of whom 15 were over 10,000 copies/ml. Among 130 and 138 patients on ART for at least 24 and 36 months, 112 (86.1%) and 134 (97.1%) patients, respectively, had undetectable VL. The overall treatment success ratio for the cohort on ART for at least 24 months was 86% in an intention-to-treat analysis. Among 22 children with a VL above 1000 copies after 24 months of ART, only two were in immunological failure according to the WHO criteria [18].

### Reverse transcriptase HIV-1 drug resistance patterns

**Figure 4:**
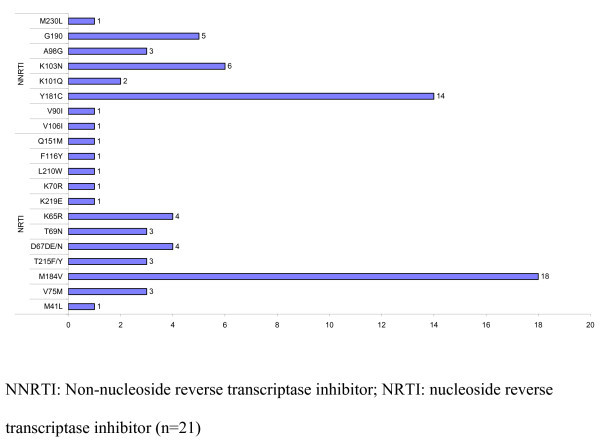
**Most frequently found mutations in patients on ART >24 months**.

HIV-1 reverse transcriptase genotyping was performed for 21 patients with VLs above 1000 copies/ml after 24 months of ART. Genetic analysis revealed that all these children were infected with CRF01_AE viruses. According to the WHO HIV epidemiological survey for scoring resistance-associated mutations list, the most frequent mutations were M184I (n = 18), Y181C (n = 15), K103N (n = 7) and G190A (n = 6) (Figure 4). All these children were found to be resistant to nevirapine/efavirenz. Seventeen of 21 were resistant to lamivudine/emtricitabine and three others possibly resistant to lamivudine/emtricitabine. Six children of 21 were resistant to stavudine and/or zidovudine. Two were resistant to didanosine and three others possibly resistant to this nucleoside reverse transcriptase inhibitor, three to abacavir and three possibly resistant to abacavir. Finally, four were resistant and one possibly resistant to tenofovir.

Six out of 22 children had developed a profile of extensive resistance, explained by the presence of the K65R mutation in four children and Q151 M in two children.

### Factors associated with a VL >1000 copies/ml

Only CD4 counts and CD4 percentages below the threshold for severe immunosupression [18] at 24 and 36 months predicted treatment failure at those times. Children coming late to their clinic appointments were not at higher risk for virological failure. Orphan status and caregiver characteristics, including literacy, age and socio-economic status, were similarly not associated with treatment failure after 24 months of ART.

## Discussion

This study, with a large cohort of children on ART, showed that first-line treatment remained effective in the medium term. Ninety-three percent of children in our HIV-programmes were alive and in care at the end of the observation period. Rapid and sustained immune restoration over time, a high virological success rate of 86% and improvement in weight-for-age z-scores were observed for children on ART for more than 24 months.

These satisfactory results offer reassuring evidence that good clinical, immunological and virological outcomes can be maintained up to two to three years after initiating ART in programmatic settings in countries like Cambodia. Overall, the clinical and immunological outcomes in this study compared favourably with published cohort studies in similar contexts [1-15] and were often better than those reported in western cohorts [20-25]. The proportion of children who achieved virological suppression was also comparable to that reported by clinical trials in high-income countries [1,25].

Among patients with detectable VLs and in whom genotyping was performed, all had at least one reverse transcriptase inhibitor-associated resistance mutation. The most common mutations were associated with resistance to non-nucleoside reverse transcriptase inhibitors and/or lamivudine, similar to other first-line cohort studies in resource-limited settings describing HIV-1 reverse transcriptase mutation patterns. In future, standardized second-line regimens based on the type of first-line treatments should be evaluated through HIV genotyping pilot studies.

Six out of 22 patients developed a profile of extensive resistance, limiting the options for choosing effective second-line ART regimens. In our clinic, a very limited number of antiretroviral (ARV) drugs were available, common to most HIV clinics in the developing world. Despite excellent short- and medium-term programme outcomes in these settings, an expanded drug formulary will eventually be needed as pediatric cohorts mature and ARV resistance becomes inevitable.

Unfortunately, drugs used for standardized second-line regimens, such as abacavir, didanosine and lopinavir/ritonavir, are still discouragingly expensive [26]. In addition, there are other problems: safe and easy-to-administer pediatric formulations are still not readily available; some drugs have yet to be tested on children (e.g., tenofovir); and some new drugs are marketed at prices that are much higher than those for equivalent adult doses [27,28]. Thus, there is an urgent need for access to affordable pediatric formulations for the developing world.

In the multivariate model, the only significant risk factor for treatment failure after 24 and 36 months of ART was a CD4 count or CD4 percentage below the threshold for severe immunosupression at those months. A recent study in Thailand has found that children who received nevirapine were 3.7 times more likely to develop virologic failure than those receiving efavirenz, but in our setting we have only few children receiving efavirenz [29].

We calculated the sensitivity, specificity and positive and negative predictive value (PPV, NPV) of the WHO immunological criteria for failure among this cohort of pediatric HIV patients. While these criteria showed a high specificity, PPV and NPV (100%, 100% and 92.9%, respectively), the sensitivity was unacceptably low at only 10%. Thus, relying exclusively on WHO immunological criteria would have led to a misclassification of 20 out of 22 treatment failures among the children in our cohort. This finding supports the importance of viral load measurement as a routine monitoring tool for ART.

In our previous 12-month study, being an orphan was found to be a greater risk for virological failure, but this factor was not seen here [3]. At that time, virological failure was hypothesized to be the result of poor adherence. As a result, greater attention was given to adherence support for this group of children and their caregivers. Intensive home-based care was provided for those staying at home with family members or neighbours. Specific counselling sessions were organized for elder and illiterate caregivers, such as grandparents and older relatives. A social worker was employed to provide support to these children and their caretakers and to link the families with organizations and institutions that provided financial assistance.

Finally, regular training sessions were provided to staff in orphanages to improve their care of the children. The current study design could not establish a causal relationship between increased support and improved adherence, but it underlined the importance of regular and systematic analyses of programme outcomes that can result in concrete action.

A very low loss-to-follow-up rate was observed in our cohort. Even though we were not able to systematically record treatment adherence as expressed by actual pill intake or evaluation of plasma ARV concentrations, we systematically recorded clinic attendance as a proxy for treatment adherence. Home-based care and early systematic tracing of defaulters was established and followed up with intensive counselling and psychosocial support for both children and their caregivers. Caretakers often reported that the clinical benefits of the treatment were rapidly apparent and that this motivated them to adhere to treatment.

These factors might explain not only the very low lost-to-follow-up rate, but also the good overall programme outcomes. Studies from similar settings suggest that higher rates of ART adherence are observed in developing countries and this holds true even for pediatric patients [12]. Meta-analyses have suggested that African children's adherence to ART is higher than their western peers, and this may be responsible for better therapeutic responses [30-32], but it has not been well documented in Asian contexts.

There are several limitations to this study. First, due to the unavailability of diagnostic tools for infants in the early years of the programme, only a few were started on ART. This probably biased the results towards a better overall outcome. Second, previous ART experience, including exposure to prevention of mother to child transmission regimens, was only self-reported in our clinic (by the caregiver) and we could not verify this. Third, we were not able to systematically record antiretroviral drug toxicities and adverse events in this programme. Fourth, the small number of treatment failures limited the power of the multivariate analysis for identifying risk factors. However, the routine system for monitoring patients, using patient files and electronic records, was robust and regularly checked by supervising teams, and we believe that the results are reliable and representative.

Despite these limitations, our study provides reassuring evidence on the medium-term effectiveness of ART in a non-urban, resource-constrained setting.

## Conclusions

Good survival, immunological restoration and viral suppression were sustained after two to three years of ART among children in resource-constrained settings. Increased access to routine virological measurements is needed for timely diagnosis of treatment failure. Improved access to tolerable, affordable pediatric formulations of second-line ART is also critical so that children in whom virologic failure is diagnosed will have treatment options available.

## Competing interests

The authors declare that they have no competing interests.

## Authors' contributions

PI and MER conceived and designed the study protocol. PI was the study coordinator. TV, CST, KA, EA and VK carried out the study and collected data in the field. SN and EN did the virological evaluation. MER did the statistical analysis. PI and RZ participated in the statistical analysis and data interpretation. PI led the writing of the manuscript; all investigators participated in its final writing and editing.
